# Phylogenetic position of Loricifera inferred from nearly complete 18S and 28S rRNA gene sequences

**DOI:** 10.1186/s40851-015-0017-0

**Published:** 2015-06-30

**Authors:** Hiroshi Yamasaki, Shinta Fujimoto, Katsumi Miyazaki

**Affiliations:** Department of Chemistry, Biology & Marine Science, Faculty of Science, University of the Ryukyus, Senbaru 1, Nishihara, Nakagami, Okinawa 903-0213 Japan; Department of Zoology, Division of Biological Science, Graduate School of Science, Kyoto University, Kitashirakawa-Oiwakecho, Sakyo-ku, Kyoto 606-8502 Japan; Seto Marine Biological Laboratory, Field Science Education and Research Center, Kyoto University, Wakayama, 649-2211 Japan

**Keywords:** Molecular phylogeny, Ecdysozoa, Scalidophora, Cycloneuralia, Nematoida, Panarthropoda

## Abstract

**Background:**

Loricifera is an enigmatic metazoan phylum; its morphology appeared to place it with Priapulida and Kinorhyncha in the group Scalidophora which, along with Nematoida (Nematoda and Nematomorpha), comprised the group Cycloneuralia. Scarce molecular data have suggested an alternative phylogenetic hypothesis, that the phylum Loricifera is a sister taxon to Nematomorpha, although the actual phylogenetic position of the phylum remains unclear.

**Methods:**

Ecdysozoan phylogeny was reconstructed through maximum-likelihood (ML) and Bayesian inference (BI) analyses of nuclear 18S and 28S rRNA gene sequences from 60 species representing all eight ecdysozoan phyla, and including a newly collected loriciferan species.

**Results:**

Ecdysozoa comprised two clades with high support values in both the ML and BI trees. One consisted of Priapulida and Kinorhyncha, and the other of Loricifera, Nematoida, and Panarthropoda (Tardigrada, Onychophora, and Arthropoda). The relationships between Loricifera, Nematoida, and Panarthropoda were not well resolved.

**Conclusions:**

Loricifera appears to be closely related to Nematoida and Panarthropoda, rather than grouping with Priapulida and Kinorhyncha, as had been suggested by previous studies. Thus, both Scalidophora and Cycloneuralia are a polyphyletic or paraphyletic groups. In addition, Loricifera and Nematomorpha did not emerge as sister groups.

**Electronic supplementary material:**

The online version of this article (doi:10.1186/s40851-015-0017-0) contains supplementary material, which is available to authorized users.

## Introduction

Since its first description as a new phylum [1], Loricifera has been one of the most enigmatic metazoan phyla. Although only 35 loriciferan species have been described worldwide, the actual species diversity is higher, as many new species await description [2–6]. All known loriciferan species are microscopic (80–800 μm) and occur in marine sediments, such as mud, sand, and shell gravel. The most extreme habitat for Loricifera is the hypersaline anoxic deep basin in the Mediterranean Sea, where members of this phylum are metabolically active [6, 7]. Our knowledge of loriciferan life cycles is also only fragmentary, given the recent findings of new life cycles and larval types [3–5, 8].

There are two alternative hypotheses on the position of Loricifera within Ecdysozoa, both based on morphological data. One is the ‘Scalidophora hypothesis’ [9–11], in which Loricifera, Kinorhyncha, and Priapulida together comprise a clade, Scalidophora. Morphological similarities between Scalidophora and Nematomorpha [12–15] and between Scalidophora and Nematoida (Nematomorpha and Nematoda) [9, 11, 16–21] have indicated that these five phyla in turn comprise a clade, Cycloneuralia [20, 21].

The alternative is the ‘Loricifera + Nematomorpha hypothesis’ [22]. While the first molecular phylogenetic study that included a loriciferan sequence (18S rRNA) failed to establish the phylogenetic position of Loricifera [23], Sørensen et al. [22] detected a sister group relationship between Loricifera and Nematomorpha based on 18S rRNA and histone-3 sequences, although with low nodal support (posterior probability = 0.83). The latter study also detected a sister group relationship between Priapulida and Kinorhyncha, but not monophyly for Cycloneuralia, which several previous molecular studies that lacked loriciferan sequences had indicated [24–29].

The present study investigated the phylogenetic position of phylum Loricifera within Ecdysozoa using nearly complete 18S and 28S rRNA sequences. Also of interest was the phylogenetic status of the taxa Scalidophora and Cycloneuralia.

## Materials and methods

### Sampling and DNA sequencing

**Fig. 1:**
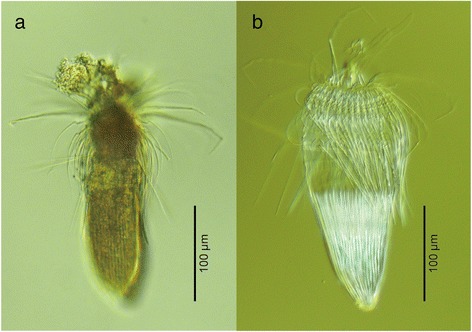
*Rugiloricus* sp., an undescribed loriciferan. Nomarski photomicrographs of the hologenophore of the specimen of *Rugiloricus* sp. used in this study. **a**, Entire animal before DNA extraction; **b**, Exoskeleton of the specimen after DNA extraction

The loriciferan specimen used in this study was collected from Ise Bay, Japan, northwestern Pacific (34°9.77′N, 136°51.40′E, 161–174 m depth) during a cruise of the TR/V Seisui-maru (Mie University) on 21 November 2013. A sediment sample was collected with a biological dredge, subsequently frozen to prevent DNA degradation, and sent to the laboratory. In the laboratory, meiofaunal specimens were extracted by floatation [30] with Ludox® HS 40. The extracted sample was sorted under a stereomicroscope, and a single adult loriciferan specimen (Fig. 1a) was obtained and preserved in 99 % EtOH for DNA extraction.

Total genomic DNA was extracted [31] from the specimen with a DNeasy Tissue Kit (Qiagen, Tokyo). After DNA extraction, the exoskeleton was mounted in Fluoromount G® as a hologenophore (Fig. 1b). The loriciferan specimen was identified as *Rugiloricus* sp. based on the morphology of the hologenophore.

Nearly complete 18S rRNA (18S) and 28S rRNA (28S) genes sequences were amplified by PCR using previously published primer sets and conditions [31]. All nucleotide sequences were determined by direct sequencing with a BigDye Terminator Kit ver. 3.1 (Life Technologies, Co., USA) and a 3730 DNA Analyzer (Life Technologies, Co., USA). Sequence fragments were assembled by using MEGA 5 [32]. After assembly, 18S (1872 bp) and 28S (3450 bp) sequences were deposited in GenBank under accession numbers LC032019 and LC032020.

### Phylogenetic analyses

18S and/or 28S sequences for 66 taxa were obtained from GenBank. We prepared the following five datasets for analyses (Table 1): “18S + 28S (50OTU)” including 18S and 28S sequences for all 50 taxa which both 18S and 28S are available (note that we treated the 18S sequence from *Milnesium tardigradum* and the 28S sequence from *Milnesium* sp. as a single OTU, because nearly complete 18S and 28S sequences were unavailable from a single tardigrade species); “18S (50OTU)” including 18S sequences for the same taxa of “18S +28S (50 OTU)”; “28S (50OTU)” including 28S sequences for the same taxa of “18S +28S (50 OTU)”; “18S (65 OTU)” including 18S sequences for more comprehensive taxon sampling especially in Tardigrada, Nematoda, Nematomorpha, Priapulida, and Kinorhyncha than the former three datasets; “18S (63 OTU)” including 18S sequences for same OTU to “18S (65 OTU)” except for *Nanaloricus* sp. due to its short sequence and *Meiopriapulus fijiensis* to avoid long branch attraction [22]. Sequences from each gene were pre-aligned separately with MAFFT software [33] using the FFT-NS-2 option and were subsequently divided into domains by eye. Domain sequences were realigned individually with MAFFT software using the L-INS-i option (Additional files 1, 2, 3 and 4). Alignment-ambiguous positions were removed with TrimAl software [34] in “strict setting”, and all positions bearing gaps were also removed. The trimmed domain sequences were recombined to form the final dataset for analysis (Additional files 5, 6, 7 and 8), which was 1426 bp long for 18S and 2189 bp long for 28S in “18S + 28S (50OTU)”, “18S (50OTU)”, and “28S (50OTU), 1277 bp long for 18S in 18S (65 OTU), and 1302 bp long for 18S in 18S (63 OTU). The chi-square test in Kakusan4 [35] indicated that the base composition of each dataset was significantly homogeneous.

**Table 1:** List of taxa included in each dataset

Taxa			Data set					Accession number	
		Species	18S + 28S (50OTU)	18S (50OTU)	28S (50OTU)	18S (65OTU)	18S (63OTU)	18S	28S
Loricifera		*Rugiloricus* sp.	○	○	○	○	○	LC032019	LC032020
		*Nanaloricus* sp.				○		EU669461	
		*Pliciloricus* sp.				○	○	AY746986	-
Arthropoda	Euchelicerata	*Limulus polyphemus*	○	○	○	○	○	U91490	AF212167
		*Calocheiridius* cf. *termitophilus*	○	○	○	○	○	AY859559	AY859558
		*Siro rubens*	○	○	○	○	○	U36998	AY859602
		*Eremobates* sp.	○	○	○	○	○	AY859573	AY859572
		*Pandinus imperator*	○	○	○	○	○	AY210831	AY210830
		*Mastigoproctus giganteus*	○	○	○	○	○	AF005446	AY859587
		*Misumenops asperatus*	○	○	○	○	○	AY210445	AY210461
	Pycnogonida	*Anoplodactylus portus*	○	○	○	○	○	AY859551	AY859550
		*Callipallene* sp.	○	○	○	○	○	AY210808	AY210807
	Myriapoda	Polyxenidae sp.	○	○	○	○	○	AY859596	AY859595
		*Orthoporus* sp.	○	○	○	○	○	AY210829	AY210828
		*Cherokia georgiana*	○	○	○	○	○	AY859563	AY859562
		*Scutigera coleoptrata*	○	○	○	○	○	AF173238	AY859601
		*Craterostigmus tasmanianus*	○	○	○	○	○	AF000774	AY859569
	Crustacea	Cyprididae sp.	○	○	○	○	○	AY210816	AY210815
		*Anaspides tasmaniae*	○	○	○	○	○	L81948	AY859549
		*Squilla empusa*	○	○	○	○	○	L81946	AY210842
		*Heteromysis* sp.	○	○	○	○	○	AY859580	AY859578–79
		*Gaetice depressus*	○	○	○	○	○	AY859577	AY859575–76
		*Panulirus argus*	○	○	○	○	○	U19182	AY210833–35
		*Homarus americanus*	○	○	○	○	○	AF235971	AY859581
		*Eulimnadia texana*	○	○	○	○	○	AF144211	AY859574
		*Triops longicaudatus*	○	○	○	○	○	AF144219	AY157606
	Hexapoda	*Podura aquatica*	○	○	○	○	○	AF005452	AY210838
		*Sminthurus viridus*	○	○	○	○	○	AY859604	AY859603
		*Dilta littoralis*	○	○	○	○	○	AF005457	AY859570–71
		*Callibaetis ferrugineus*	○	○	○	○	○	AF370791	AY859557
		*Mantis religiosa*	○	○	○	○	○	AY859586	AY859585
		*Zootermopsis angusticollis*	○	○	○	○	○	AY859615	AY859614
		*Gromphadorhina laevigata*	○	○	○	○	○	AY210820	AY210819
		Gomphocerinae sp.	○	○	○	○	○	AY859547	AY859546
		*Vespula pensylvanica*	○	○	○	○	○	AY859613	AY859612
		*Merope tuber*	○	○	○	○	○	AF286287	DQ202351
Onychophora		*Peripatoides novaezealandiae*	○	○	○	○	○	AF342794	AF342791–93
Tardigrada		*Milnesium tardigradum*	○	○		○	○	U49909	-
		*Milnesium* sp.	○		○			-	AY210826
		*Echiniscus blumi*				○	○	HM193375	
		*Testechiniscus spitzbergensis*				○	○	EU266967	
		*Richtersius coronifer*				○	○	AY582123	
Nematoda	Spiurina	*Ascaris lumbricoides*	○	○	○	○	○	U94366	AY210806
	Dorylaimia	*Trichinella spiralis*	○	○	○	○	○	U60231	AF342803
		*Xiphinema rivesi*	○	○	○	○	○	AF036610	AY210845
	Enoplia	*Pontonema vulgare*				○	○	AF047890	
	Desmodorida	*Spirinia elongata*				○	○	EF527426	
	Monhysterida	*Theristus agilis*				○	○	AY284695	
Nematomorpha		*Chordodes morgani*	○	○	○	○	○	AF036639	AF342787
		*Gordius aquaticus*	○	○	○	○	○	X80233	AY210817
		*Nectonema agile*				○	○	AF421767	
Priapulida		*Priapulus caudatus*	○	○	○	○	○	Z38009	AY210840
		*Halicryptus spinulosus*	○	○	○	○	○	AF342790	AF342789
		*Tubiluchus corallicola*				○	○	AF119086	
		*Meiopriapulus fijiensis*				○		JN211192	
Kinorhyncha		*Pycnophyes* sp.	○	○	○	○	○	AY859598	AY859597
		*Dracoderes abei*				○	○	AB738350	AB738351
		*Echinoderes dujardinii*				○	○	LC007044	LC007065
		*Centroderes spinosus*				○	○	KF372858	
		*Campyloderes* cf. *vanhoeffeni*				○	○	LC007037	
Lophotrochozoa (Outgroup)	Nemertea	*Amphiporus* sp.	○	○	○	○	○	AF119077	AF342786
	Mollusca	*Placopecten magellanicus*	○	○	○	○	○	X53899	AF342798
	Platyhelminthes	*Stylochus zebra*	○	○	○	○	○	AF342801	AF342800
	Echiura	*Urechis caupo*	○	○	○	○	○	AF342805	AF342804
Deuterostomes (Outgroup)	Hemichordata	*Ptychodera fava*	○	○	○	○	○	AF278681	AF212176
	Chordata	*Ciona intestinalis*	○	○	○	○	○	AB013017	AF212177

Taxa included in each data set, with GenBank accession numbers for sequences

Before the analyses, the optimal substitution model was determined with Kakusan4 to be the general time-reversible model with the gamma distribution (GTR + Γ). Phylogenetic trees were constructed by maximum likelihood (ML) implemented in raxmlGUI 1.2 [36, 37], and Bayesian inference (BI) implemented in MrBayes 3.2.1 [38, 39]. Nodal support for the ML tree was assessed through analyses of 1000 bootstrap pseudoreplicates. For BI, Markov-chain Monte-Carlo searches were performed with four chains, each of which was run for 1,000,000 generations, with trees sampled every 100 generations. Stationarity was evaluated by monitoring likelihood values graphically. The initial 20 % of trees from each run were discarded as burn-in, and the remaining trees were used to construct majority-rule consensus trees and determine the Bayesian posterior probability for each clade [39].

## Results and discussion

### Overall topology in Ecdysozoa

**Fig. 2:**
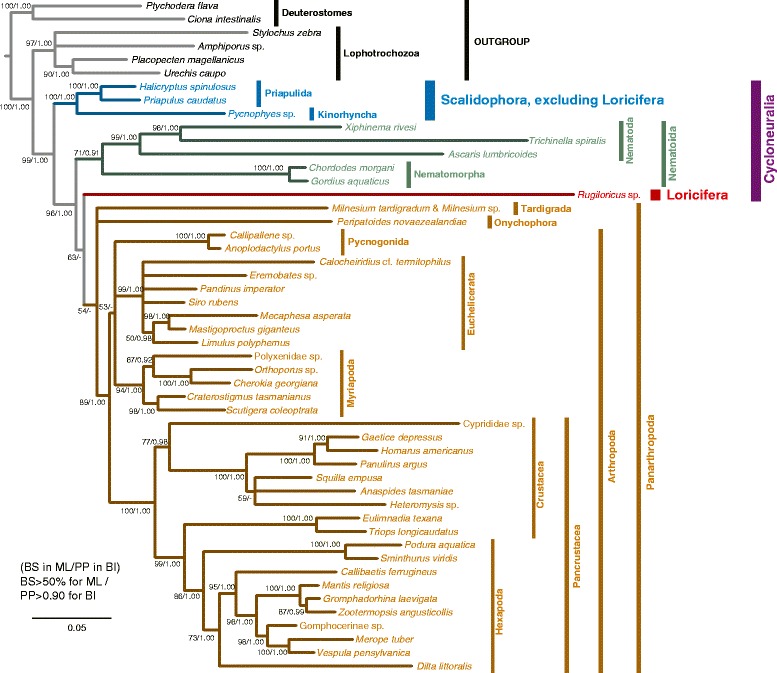
Maximum-likelihood tree of 18S + 28S (50 OTU) dataset. The tree is based on 18S + 28S (50 OTU) dataset. Numbers near nodes are the maximum-likelihood bootstrap (BS) and Bayesian posterior probability (PP) values, respectively; values lower than 50 % (BS) or 0.90 (PP) are indicated by dashes. The scale bar indicates branch length in substitutions per site

None of the trees conflicted with the others in their overall topology; however, supporting values were lower in datasets with more OTU and shorter sequences (Table 2; Additional files 9, 10, 11 and 12). In our results, increasing the available sequence length with slightly limited taxa generated a better-resolved tree than using more taxa with markedly shortening the sequence length. Thus, we present and mainly discuss the result of 18S + 28S (50 OTU) dataset (Fig. 2). Both the ML and BI trees showed monophyly for the Ecdysozoa (nodal support ML/PP = 99/1.00) as well as for the phyla Priapulida (100/1.00), Nematoda (99/1.00), Nematomorpha (100/1.00), and Arthropoda (89/1.00). Although the monophyly of each phyla were not tested for Kinorhyncha, Loricifera, and Tardigrada in 18S + 28S (50 OTU) dataset, they were supported in 18S (65 OTU) and 18S (63 OTU) with the maximum supporting values (Table 2). Monophyly for Onychophora was not tested due to the inclusion of a single representative of the phylum in all datasets.

**Table 2:** Summary of the results of each dataset

Clade	supporting value (ML/BI)				
	18S + 28S (50 OTU)	28S (50 OTU)	18S (50 OTU)	18S (65 OTU)	18S (63 OTU)
Ecdysozoa	99/1.00	71/0.99	94/1.00	89/1.00	88/1.00
Priapulida + Kinorhyncha	100/1.00	96/1.00	89/1.00	-/0.93	76/0.99
Nematoida + Loricifera + Panarthropoda	96/1.00	72/0.99	-/0.90	−/−	-/0.95
Nematoida	71/0.91	50/-	-/0.91	−/−	−/−
Loricifera + Panarthropoda	63/-	75/0.95	−/−	−/−	−/−
Panarthropoda	54/-	54/-	−/−	−/−	−/−
Priapulida	100/1.00	100/1.00	96/1.00	−/−	98/1.00
Kinorhyncha	−/−	−/−	−/−	100/1.00	100/1.00
Nematoda	99/1.00	85/1.00	91/1.00	77/1.00	61/0.99
Nematomorpha	100/1.00	100/1.00	100/1.00	96/1.00	95/1.00
Loricifera	−/−	−/−	−/−	100/1.00	100/1.00
Tardigrada	−/−	−/−	−/−	100/1.00	100/1.00
Arthropoda	89/1.00	82/1.00	−/−	−/−	-/0.98
**Nematoda + Tardigrada + Arthropoda**	**−/−**	**−/−**	**−/−**	**-/0.98**	**-/0.91**
**Tardigrada + Arthropoda**	**−/−**	**−/−**	**-/0.90**	**−/−**	**−/−**
**Nematoda + Tardigrada**	**−/−**	**−/−**	**−/−**	**−/−**	**-/0.93**

Summary of the results of analyses based on each dataset. Reconstructed clades with supporting values (maximum-likelihood bootstrap/Bayesian posterior probability) in each dataset are listed. Supporting values lower than 50 % (bootstrap values) or 0.90 (posterior probability) are considered as nonsignificant and indicated by dashes. Dark highlighted clades are supported only in Bayesian tree of short-sequence datasets, 18S (50 OTU), 18S (65 OTU), and 18S (63 OTU), thus these clades are not regarded as actual clades

Within the Ecdysozoa, two basal clades were detected with high nodal support: Priapulida + Kinorhyncha (Scalidophora, excluding Loricifera; nodal support 100/1.00) and Nematoda + Nematomorpha + Loricifera + Tardigrada + Onychophora + Arthropoda (99/1.00). The latter basal clade in turn comprised the clades Nematoda + Nematomorpha clade (= Nematoida), and Loricifera + Tardigrada + Onychophora + Arthropoda clade (= Loricifera + Panarthropoda) in both the ML and BI trees. Support for the Nematoida clade was only moderate (71/0.90), and that for Loricifera + Panarthropoda clade was low (63/0.66). Support for the monophyly of Tardigrada + Onychophora + Arthropoda (= Panarthropoda) was also low (54/0.76). Tardigrada, Onychophora, and Arthropoda formed an unresolved trichotomy.

### Phylogenetic evaluation of loricifera, scalidophora, and cycloneuralia

The clade we detected consisting of Loricifera, Nematoida, and Panarthropoda received high nodal support (96/1.00), but the phylogenetic position of Loricifera within this clade remains unclear, as support for the node grouping Loricifera with Panarthropoda was quite low (63/0.66). However, the scalidophoran phyla Priapulida and Kinorhyncha together comprised a clade with high nodal support (100/1.00) to the exclusion of Loricifera, which instead grouped in a highly supported (96/1.00) clade with Nematoida and Panarthropoda. Our results thus do not support both the ‘Scalidophora hypothesis,’ in which Loricifera comprises a clade with Kinorhyncha and Priapulida, and the ‘Loricifera + Nematomorpha hypothesis’. Our trees also indicated non-monophyly for Cycloneuralia, as Loricifera and Nematoida showed closer relationships to Panarthropoda than to other cycloneuralian phyla (Priapulida and Kinorhyncha).

### Evaluation of synapomorphies for scalidophora and cycloneuralia

Morphological synapomorphies have previously been proposed that uniting the scalidophoran phyla (Loricifera, Priapulida and Kinorhyncha) and the cycloneuralian phyla (Scalidophora plus Nematoda and Nematomorpha). Putative synapomorphies [11] among Loricifera, Priapulida, and Kinorhyncha include (1) an introvert that has short, spinose scalids that are staggered in arrangement and triradiate in cross-section, and that has (2) inner and outer retractor muscles; (3) a compound filter of protonephridia consisting of two or more terminal cells; (4) basally thickened cuspidate spines; and (5) sensory organs (flosculi) with external cuticular micropapillae and a central pore. The most important synapomorphy proposed for cycloneuralians is the collar-shaped circumoral brain consisting of a ring neuropil [20, 21]. Our results failed to support the monophyly of either Scalidophora or Cycloneuralia, and the putative synapomorphies supporting these groups thus need to be reevaluated.

**Fig. 3:**
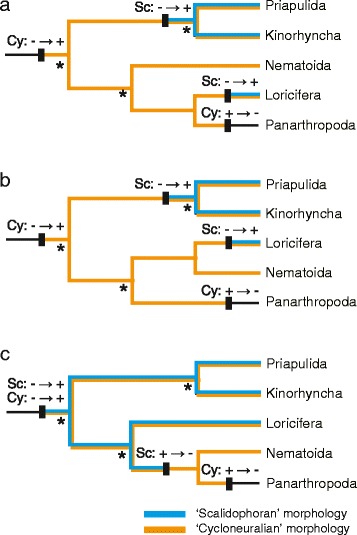
Hypotheses of evolutionary transitions in scalidophoran and cycloneuralian morphological characters. These hypotheses are based on the three possible relationships within the Loricifera + Nematoida + Panarthropoda clade. Sc and Cy above branches indicate morphological characters of the ‘Scalidophora’ and ‘Cycloneuralia,’ respectively; ‘ + ’ and ‘–’ indicate the presence and absence of characters; asterisks indicate well-supported nodes

With regard to the monophyly of Loricifera + Nematoida + Panarthropoda that we detected, three possible topologies among these groups (Fig. 3) in turn suggest two possible evolutionary scenarios for the three scalidophoran phyla (Priapulida, Kinorhyncha, Loricifera). If Loricifera is the sister taxon of Panarthropoda (Fig. 3a) or of Nematoida (Fig. 3b), the most parsimonious scenario is that ‘scalidophoran’ characters arose independently in Loricifera and in the common ancestor of Priapulida + Kinorhyncha and represent convergent characters. Alternatively, if Loricifera is basal in the Loricifera + Nematoida + Panarthropoda clade (Fig. 3c), the most parsimonious interpretation is that the common ancestor of Ecdysozoa possessed ‘scalidophoran’ characters, which the common ancestor of Nematoida and Panarthropoda subsequently lost.

In all three topologies (Fig. 3), the most parsimonious evolutionary scenario for ‘cycloneuralian’ characters is that they originated once in the common ancestor of Ecdysozoa and were lost once in the common ancestor of Panarthropoda. In other words, the ‘cycloneuralian’ characters are plesiomorphic in ecdysozoans.

## Conclusions

We reconstructed the phylogeny of ecdysozoan phyla using nearly complete 18S and 28S rRNA gene sequences, and our results suggested a new hypothesis for the phylogenetic position of Loricifera. These results did not support the previously proposed ‘Scalidophora’ or the ‘Loricifera + Nematomorpha’ clades, but detected a ‘Loricifera + Nematoida + Panarthropoda’ clade with rather high nodal support. Cycloneuralia emerged as paraphyletic, with high nodal support. Relationships among phyla in the ‘Loricifera + Nematoida + Panarthropoda’ clade were not well resolved, and phylogenetic analysis using transcriptomic or genomic data will be necessary to reconstruct the relationships within this clade, and to elucidate evolutionary transitions within Ecdysozoa.

### Availability of supporting data

The data sets supporting the results of this article are included within the article and its additional files.

## Additional files

Additional file 1:
**Raw 18S sequence alignment for 18S + 28S (50 OTU) and 18S (50 OTU) datasets.** Aligned 18S sequences from 50 species (44 ecdysozoan and six outgroup species) before the removal of alignment-ambiguous positions and gaps. Additional file 2:
**Raw 28S sequence alignment for 18S + 28S (50 OTU) and 28S (50 OTU) datasets.** Aligned 28S sequences from 50 species (44 ecdysozoan and six outgroup species) before the removal of alignment-ambiguous positions and gaps. Additional file 3:
**Raw 18S sequence alignment for 18S (65 OTU) dataset.** Aligned 18S sequences from 65 species (59 ecdysozoan and six outgroup species) before the removal of alignment-ambiguous positions and gaps. Additional file 4:
**Raw 18S sequence alignment for 18S (63 OTU) dataset.** Aligned 18S sequences from 63 species (57 ecdysozoan and six outgroup species) before the removal of alignment-ambiguous positions and gaps. Additional file 5:
**Final 18S sequences for 18S + 28S (50 OTU) and 18S (50 OTU) datasets.** Aligned 18S sequences of 50 species after the removal of alignment-ambiguous positions and gaps. Additional file 6:
**Final 28S sequences for 18S + 28S (50 OTU) and 18S (50 OTU) datasets.** Aligned 28S sequences of 50 species after the removal of alignment-ambiguous positions and gaps. Additional file 7:
**Final 18S sequences for 18S (65 OTU) dataset.** Aligned 18S sequences of 65 species after the removal of alignment-ambiguous positions and gaps. Additional file 8:
**Final 18S sequences for 18S (63 OTU) dataset.** Aligned 18S sequences of 63 species after the removal of alignment-ambiguous positions and gaps. Additional file 9:
**Maximum-likelihood tree of 18S (50 OTU) dataset.** The tree is based on 18S (50 OTU) dataset. Labelling of values is as in Figure 2. Additional file 10:
**Maximum-likelihood tree of 28S (50 OTU) dataset.** The tree is based on 28S (50 OTU) dataset. Labelling of values is as in Figure 2. Additional file 11:
**Maximum-likelihood tree of 18S (65 OTU) dataset.** The tree is based on 18S (65 OTU) dataset. Labelling of values is as in Figure 2. Additional file 12:
**Maximum-likelihood tree of 18S (63 OTU) dataset.** The tree is based on 18S (63 OTU) dataset. Labelling of values is as in Figure 2.
