# Supplementary figures and images for: Phylogenetic position of Loricifera inferred from nearly complete 18S and 28S rRNA gene sequences
Source: Zoological Lett. 2015 Jun 30;1:18. doi: 10.1186/s40851-015-0017-0 (PMC4657359; doi:10.1186/s40851-015-0017-0)

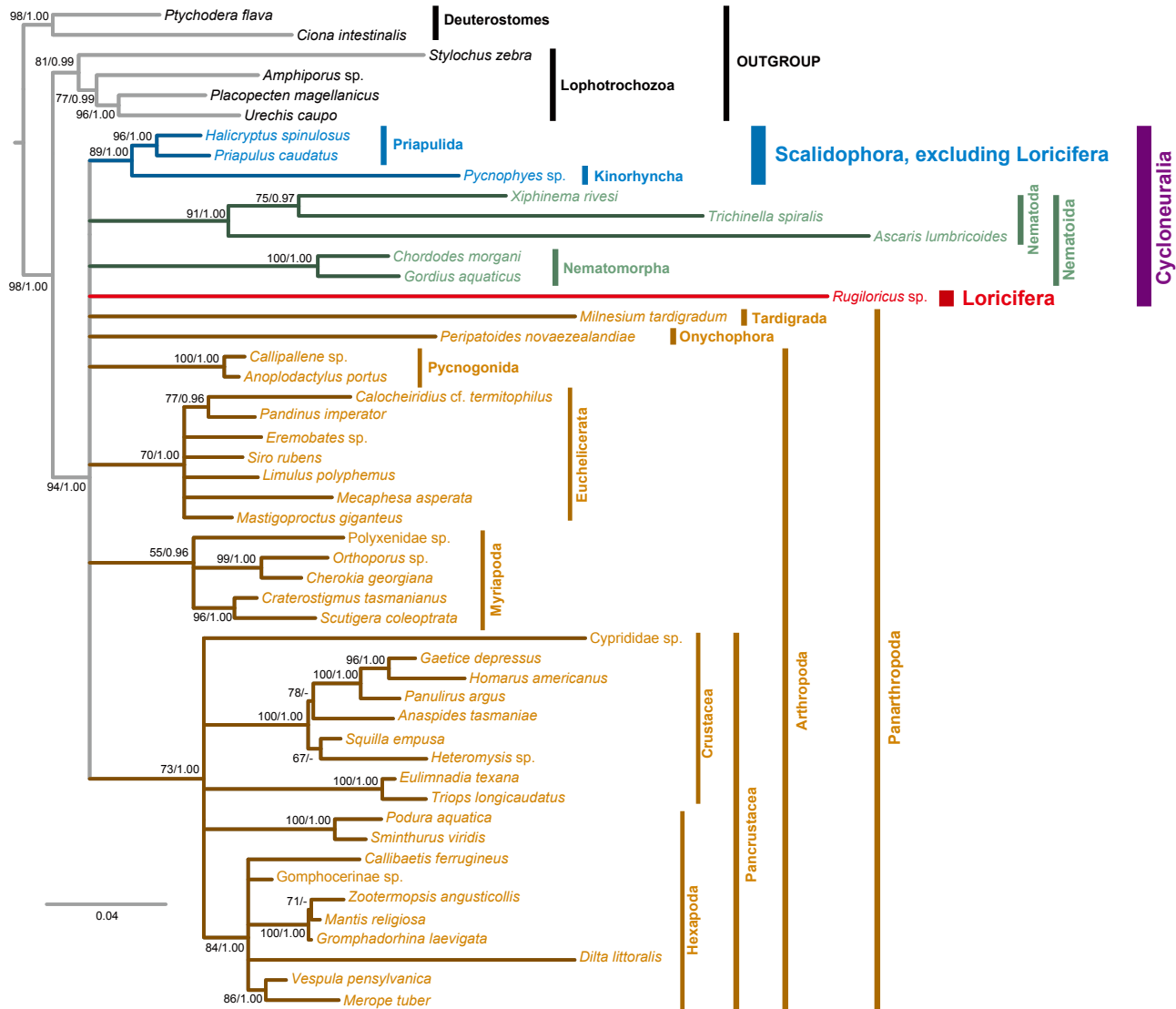

Supplement: Additional file 9: — Maximum-likelihood tree of 18S (50 OTU) dataset. The tree is based on 18S (50 OTU) dataset. Labelling of values is as in Figure 2. [file 40851_2015_17_MOESM9_ESM.pdf]

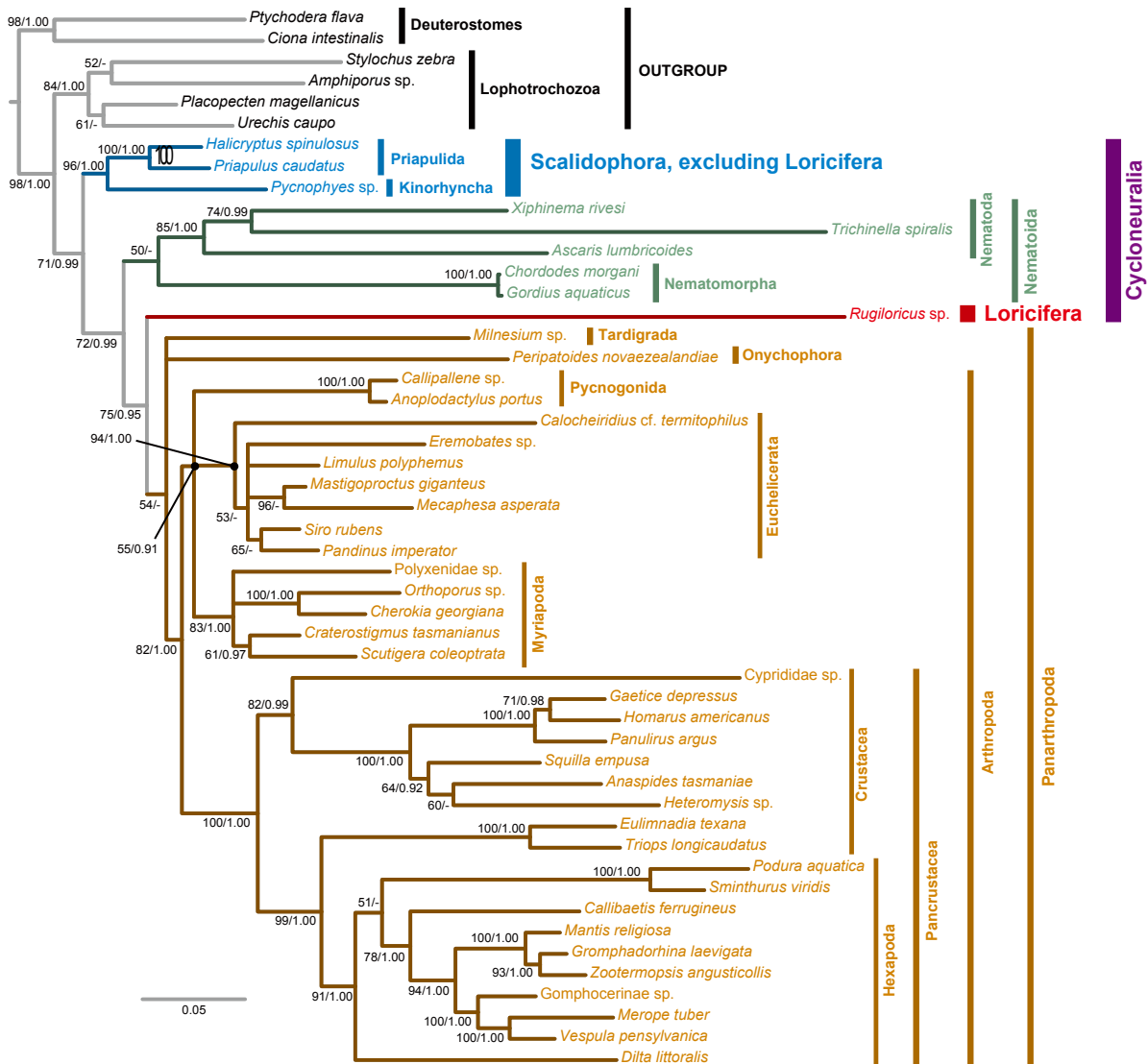

Supplement: Additional file 10: — Maximum-likelihood tree of 28S (50 OTU) dataset. The tree is based on 28S (50 OTU) dataset. Labelling of values is as in Figure 2. [file 40851_2015_17_MOESM10_ESM.pdf]

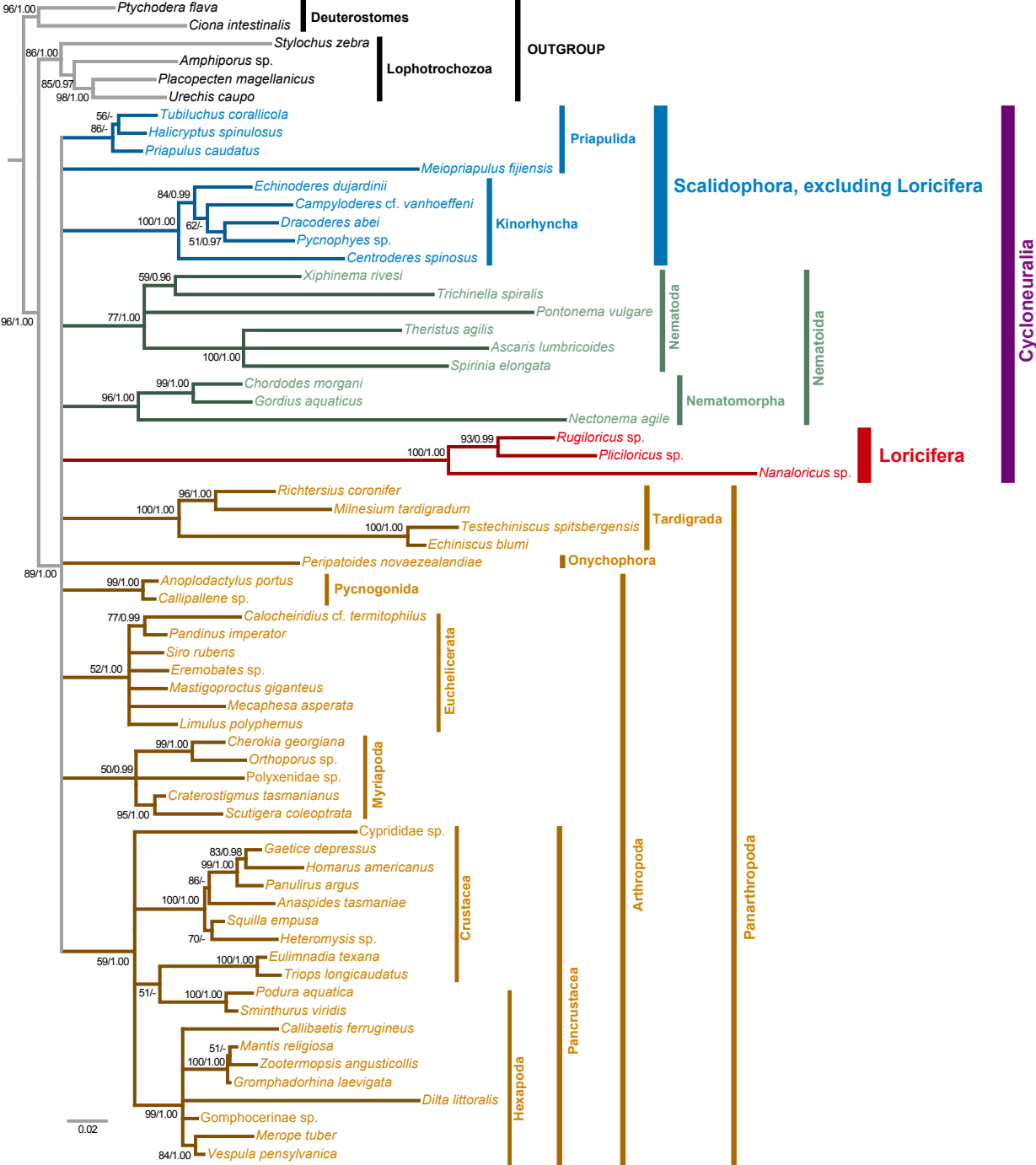

Supplement: Additional file 11: — Maximum-likelihood tree of 18S (65 OTU) dataset. The tree is based on 18S (65 OTU) dataset. Labelling of values is as in Figure 2. [file 40851_2015_17_MOESM11_ESM.pdf]

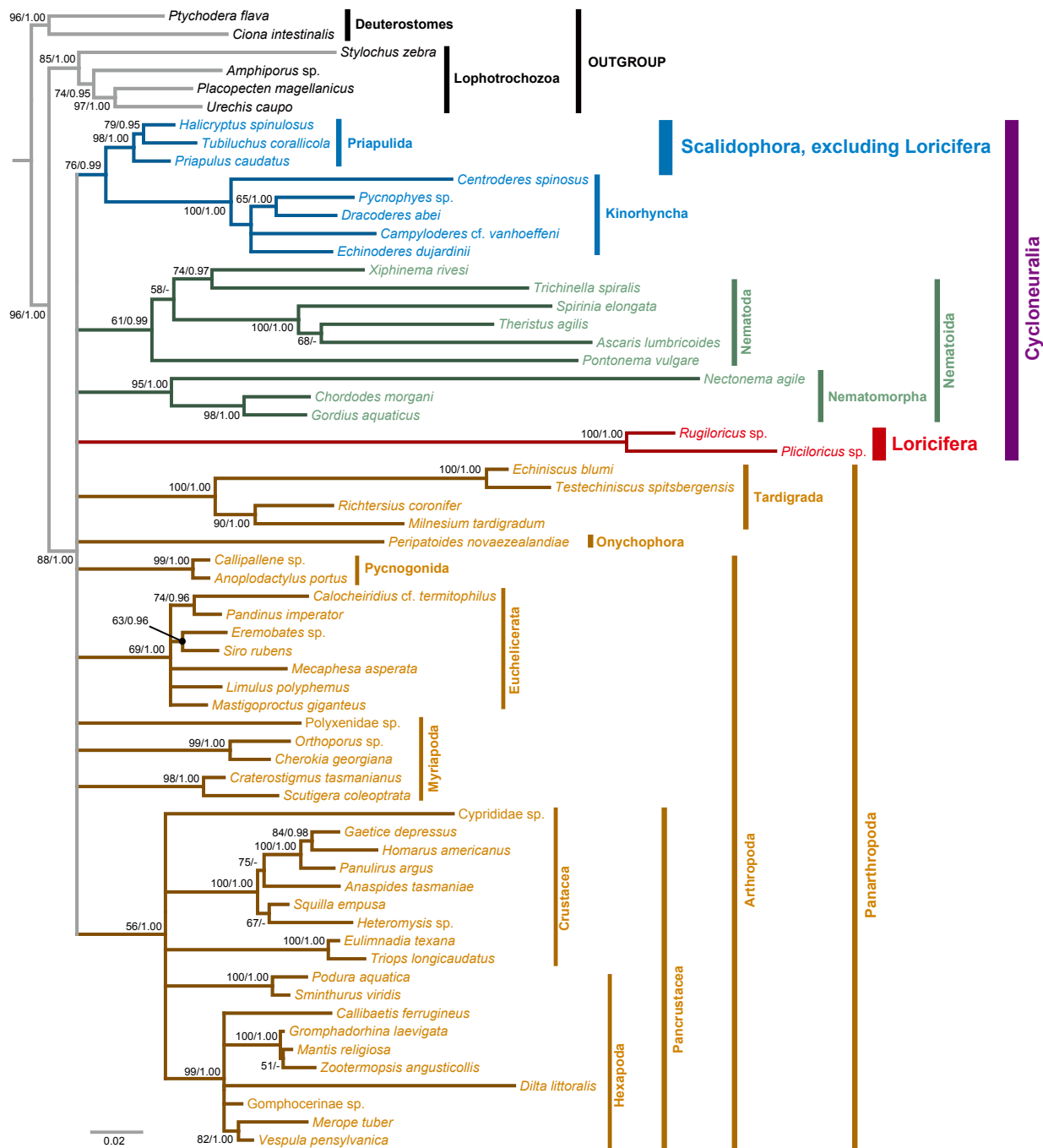

Supplement: Additional file 12: — Maximum-likelihood tree of 18S (63 OTU) dataset. The tree is based on 18S (63 OTU) dataset. Labelling of values is as in Figure 2. [file 40851_2015_17_MOESM12_ESM.pdf]
